# A review of potential health risks of radiofrequency electromagnetic field exposure from mobile phones and base stations in children and pregnant women

**DOI:** 10.3389/fpubh.2026.1915745

**Published:** 2026-09-11

**Authors:** Shivani Dogra, Sonali Rajput, Aanchal Rathi, Rohit Gautam, Poulami Mazumder, Abhinav Sinha, Rakesh Kumar Sahoo, Sanghamitra Pati, Aparna Mukherjee, Taruna Arora

**Affiliations:** 1 Division of Reproductive, Child Health & Nutrition, Indian Council of Medical Research, New Delhi, India; 2 Department of Obstetrics and Gynaecology, All India Institute of Medical Science, New Delhi, India; 3 ICMR-National Institute of Health Research, Bhubaneswar, Odisha, India

**Keywords:** base stations, birth outcomes, children, mobile phone, pregnant women, radiofrequency electromagnetic fields (RF-EMF)

## Abstract

Over the last decade, the rapid expansion of mobile communication technologies has substantially increased population exposure to radiofrequency electromagnetic fields (RF-EMF). This has raised concerns about possible health implications, particularly for vulnerable groups like children and pregnant women. Although international agencies have reported no consistent evidence of harmful effects at established exposure limits, uncertainties remain regarding long-term and developmental effects. This review synthesizes and evaluates epidemiological and experimental evidence published between 2015 and 2025; a period characterized by rapid technological transitions (4G to 5G), advances in exposure assessment techniques, and increasing reliance on wireless devices. Confining the timeframe ensures a more relevant and up-to-date assessment of current exposure patterns and emerging biological insights. The review explores the association between RF-EMF exposure and major health outcomes, including reproductive and developmental effects, cancer risk, and neurological changes. It also integrates mechanistic pathways involving oxidative stress, DNA damage, and ROS signalling. Although no definitive causal relationship has been established, the evidence remains inconsistent due to variability in study design, exposure metrics, and outcome definitions. Notably, experimental and high-exposure studies provide biologically plausible mechanisms, particularly through oxidative stress pathways; however, these findings have not been consistently observed in the human population. Children and foetuses may exhibit greater susceptibility because of developmental and physiological factors, yet high-quality longitudinal evidence remains limited. Overall, further standardized, mechanistic, and long-term studies are needed to strengthen the scientific evidence base and inform public health policy.

## 1 Introduction

Over the past three decades, mobile communication technologies have expanded rapidly, transitioning from second-generation (2G) systems to advanced fifth-generation (5G) networks worldwide (1). This progression has led to a substantial increase in data transmission speeds and improved connectivity. As a result, the population across nearly all sectors experiences continuous exposure to RF-EMF from both mobile phones and base stations. The World Health Organization has emphasized the need for further research due to increasing long-term exposure. This classification is based on the International Agency for Research on Cancer (IARC) designation of RF-EMF as a Group 2B possible human carcinogen, based on limited evidence in humans (2, 3). RF-EMF consists of non-ionizing electromagnetic waves in the frequency range of 100 kHz–300 GHz emitted by wireless technologies such as mobile phones (3G, 4G, 5G) and their associated base stations. Exposure from base stations is typically characterized as far-field, characterized by continuous, low-intensity radiation. Environmental radiofrequency (RF) power density levels in typical indoor and urban environments generally range from 0.001 to 0.1 W/m^2^. These are below the limits recommended by the International Commission on Non-Ionizing Radiation Protection (ICNIRP) in their 2020 guidelines (4). These exposures have become an unavoidable component of daily life, due to the widespread deployment of telecommunications infrastructure (5, 6).

Population exposure to RF-EMF is influenced by factors such as device exposure frequency, duration of use, and proximity to the body (7, 8). Due to widespread access to mobile phone technologies, children are often exposed at an early age and for prolonged periods (9). For instance, a high proportion of children and adolescents in countries such as UK (83%), US (95%) and Germany (54%) use or own mobile phone devices (10, 11). These trends have raised concerns about potential health effects, including cancer risk, neurodevelopmental and behavioural changes, oxidative stress, cellular damage, and sleep disturbances. Due to anatomical and physiological differences such as higher tissue conductivity and thinner skulls, children may be more susceptible to RF-EMF exposure (12, 13). However, evidence remains inconclusive as to whether children consistently absorb higher levels of RF energy than adults. Pregnant women exposed to RF-EMF represent another sensitive group, as exposure may affect foetal development. Studies have suggested adverse outcomes such as preterm birth, miscarriage (spontaneous abortion), stillbirth, congenital anomalies, and SGA (small for gestational age), including low birth weight at term as an indicator of intrauterine growth restriction (4, 14). Continuous low-level exposure from mobile phones and environmental sources may lead to prolonged foetal exposure, potentially influencing long-term health. However, epidemiological evidence specifically addressing such effects from base stations is still limited, largely due to challenges in accurately assessing exposure levels in the general population.

Many studies have shown an association between RF-EMF exposure and outcomes such as cancer risk, neurodevelopmental changes, and reproductive effects (15–17). However, the detailed understanding of the underlying biological mechanisms remains limited. In addition, multiple confounding factors influence disease development, making causal interpretation challenging. Mechanistic approaches focusing on oxidative stress, DNA damage, and endocrine alterations provide valuable insights into potential pathways through which RF-EMF exposure may contribute to adverse health effects. Recent evidence also indicates that RF-EMF exposure is constant and derived from multiple sources (smart home devices, wearable technology, and Wi-Fi routers). It highlights the need for population-based assessment approaches which are necessary to understand real-life exposure scenarios (18, 19). This review is based on published literature investigating the association between RF-EMF exposure and adverse health outcomes with a specific focus on children and pregnant women. The aim of this review is to evaluate key mechanistic pathways such as oxidative stress induction, DNA damage and endocrine modulation in relation to health outcomes including cancer risk, neurological effects, and reproductive outcomes. The summarized version of the methodology and conceptual framework is given in Table 1.

**Table 1:** Conceptual framework of RF-EMF exposure, population, and associated health outcomes.

Component	Description
Population	Children, adolescents and pregnant women from the general population
Exposure sources	Mobile phones and base stations
Exposure characteristics	Near-field and far-field exposure; duration, frequency, and intensity of use
Exposure assessment methods	Specific absorption rate (SAR), personal dosimetry, environmental measurements, self-reported usage
Primary health outcomes	Risk of cancer, neurological and neurocognitive effects (including developmental outcomes, sleep disturbances) and reproductive outcomes
Study types considered	Case–control studies and cohort studies, systematic reviews and meta-analyses, reviews based on evidence and state of science
Scientific databases used	PubMed, Web of Science, Embase, and Scopus

### 1.1 Review methodology and literature identification

This narrative review was designed to provide a targeted synthesis of epidemiological, experimental, mechanistic and review evidence on radiofrequency electromagnetic field (RF-EMF) exposure from mobile phones, base stations and related wireless technologies in children and pregnant women. A comprehensive literature search was conducted for publications published during the last 10 years from January 2015 to December 2025 across four electronic databases: PubMed, Web of Science, Embase, and Scopus. The search strategy combined controlled vocabulary with free-text keywords using Boolean operators (AND/OR) to maximise retrieval of relevant studies. Keywords related to mobile phones, mobile towers, mobile base stations, radiofrequency electromagnetic fields (RF-EMF), and health-related outcomes were used. The representative search terms were: (“radiofrequency electromagnetic field” OR “RF-EMF” OR “radiofrequency radiation”) AND (“mobile phone” OR smartphone OR “base station” OR “cell tower” OR Wi-Fi) AND (“child” OR adolescent OR pregnancy OR pregnant OR fetus OR prenatal) AND (cancer OR neurological OR cognitive OR sleep OR reproductive OR developmental).

The main evidence synthesis was restricted to last 10 years publications from January 2015 to December 2025 to capture contemporary evidence in the 4G and emerging 5G era. Human observational studies, experimental studies, systematic reviews, meta-analyses and relevant mechanistic studies were considered if they directly addressed RF-EMF exposure and the population or outcomes of interest. Studies on other ranges of electromagnetic frequency were not included as core evidence of RF-EMF unless identified specifically as contextual or mechanistic evidence. Such studies are explicitly labelled when retained. Older references were cited only when needed to describe basic biological concepts, historical evidence, exposure standards, or landmark epidemiological studies. Studies were chosen based on their relevance to the review objectives, target population, exposure characteristics, health outcomes, methodological quality and contribution to interpretation of the present evidence.

## 2 RF-EMF exposure characteristics and biophysical parameters

Biophysical parameters and exposure regimes collectively determine the distribution, transmission and absorption of electromagnetic energy in biological systems. To evaluate potential health outcomes, it is essential to understand the characteristics of the exposure source and its interaction with the human body. RF-EMF exposure is broadly categorized into near-field and far-field regions, primarily based on the distance between the source and the individual (20). Near-field exposure occurs when an individual is located within approximately one wavelength of an emitting source. This scenario is most relevant for personal devices such as mobile phones, where energy absorption is highly localized in specific body regions (e.g., head, ear, or hands). In this region, the electric and magnetic fields are non-uniform and vary rapidly in space, making exposure highly dependent on device orientation, device characteristics and user behaviour (21). The amount of energy absorbed by the biological tissues is quantified by using the Specific Absorption Rate (SAR), which is given by:

$$\mathrm{SAR}=\sigma E^{2}/\rho ,$$

where, *σ* denotes the conductivity of the tissue (S/m), *E* represents the electric field strength (V/m), and *ρ* is the tissue density (Kg/m^3^). *SAR* is expressed in watts per kilogram (W/kg), and is widely used for evaluating localised exposure in safety standards (22–24). In contrast, far-field exposure occurs at distances greater than one wavelength from the source, such as emissions from base stations or broadcast antennas (25). In this region, electromagnetic waves propagate as plane waves, and the electric and magnetic fields are perpendicular and uniformly distributed. Exposure is typically assessed using power density (P_d_), which can be expressed as:

$$(P_{d})=E^{2}/377.$$

where, *E* is the electric field strength (V/m), and 377 ohms represents the intrinsic impedance of free space. Although, far-field exposure levels are generally lower than near-field exposure, they tend to be more continuous and widespread. Consequently, population-based studies often focus on far-field exposure, as it reflects ambient environmental conditions experienced by larger populations.

SAR is particularly important for assessing RF-EMF exposure from devices used in close proximity to the body such as mobile phones, where energy absorption is localised. To prevent excessive tissue heating, regulatory bodies have established SAR limits (26, 27). For example, localized exposure SAR limits range between 1.6–2.0 W/kg depending on the applicable standard (28). Despite its usefulness, applying SAR values to real-world conditions remains challenging, particularly in epidemiological studies where exposure varies across time, usage patterns and individuals (29). Guidelines issued by the International Commission on Non-Ionizing Radiation Protection (ICNIRP) serve as the primary scientific basis for RF-EMF exposure limits worldwide, and most countries adopt these recommendations when establishing national regulations, including permissible power density levels. According to ICNIRP guidelines, exposure limits are defined using specific absorption rate (SAR) to prevent thermal effects, with a whole-body average SAR of 0.4 W kg^−1^ (30 min) for occupational exposure and a more stringent limit of 0.08 W kg^−1^ for the general public, incorporating a safety reduction factor. Additionally, compliance requires adherence to both whole-body and localized exposure limits, along with reference levels for electric and magnetic fields or incident power density across different frequency ranges, ensuring comprehensive protection under varying exposure conditions (4).

## 3 Biological mechanisms of RF-EMF interaction

**Figure 1:**
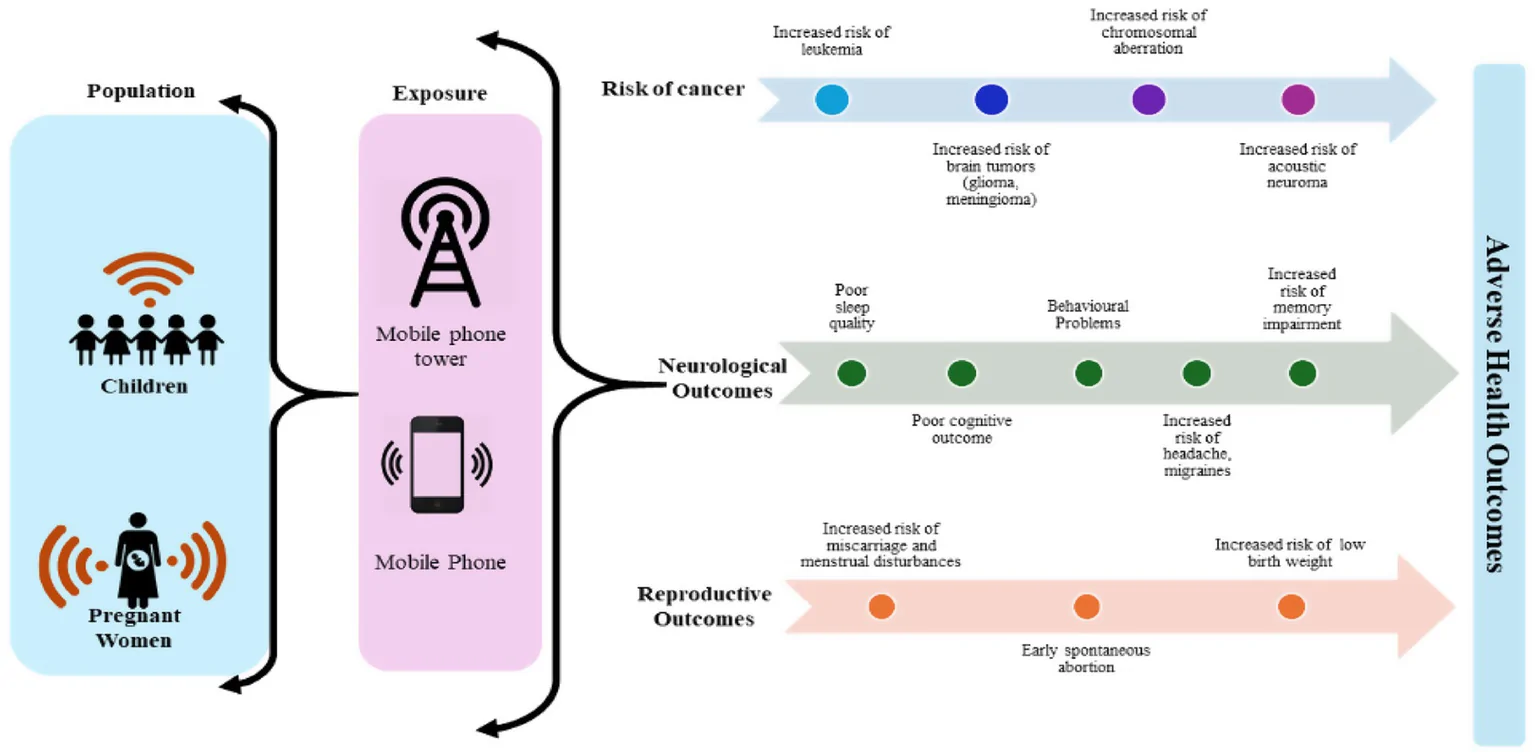
Health effects associated with mobile phone and mobile tower radiation.

Researchers have examined in detail how radiofrequency electromagnetic fields (RF-EMF) interact with living things. They have suggested that these interactions can have both thermal and non-thermal effects. Thermal effects are well-established and form the basis of current safety guidelines, but non-thermal mechanisms are still being studied and debated by scientists (Figure 1).

### 3.1 Thermal effects

When biological tissues absorb RF-EMF energy, it causes thermal effects that raise tissue temperature. The dielectric properties of tissues largely govern this process, and the Specific Absorption Rate (SAR) quantifies it (9, 30). At sufficiently elevated exposure levels, RF-EMF can induce measurable tissue heating, potentially leading to physiological alterations including protein denaturation, altered enzyme activity, and compromised cellular function. These effects are well-documented and form the scientific basis for international exposure limits established by regulatory bodies such as the International Commission on Non-Ionizing Radiation Protection (ICNIRP) (4). Under typical environmental and mobile phone usage conditions, RF-EMF exposure levels are generally below the threshold required to induce significant thermal damage. However, localized heating, particularly in tissues with limited heat dissipation (e.g., the head during prolonged mobile phone use), remains an area of investigation (31). Recent experimental and computational studies (2023–2024) have further refined thermal modelling using advanced anatomical simulations, confirming that while thermal effects are unlikely under standard exposure conditions, they remain relevant in high-intensity or occupational exposure scenarios (32–34).

### 3.2 Non-thermal effects

In contrast to thermal mechanisms, non-thermal effects refer to biological changes occurring at exposure levels that do not cause significant temperature increases. These mechanisms are less understood and remain controversial due to inconsistent findings across studies. In this review, we aimed to critically explain and summarize the proposed non-thermal mechanisms and their potential biological implications.

## 4 Carcinogenic potential of RF-EMF

Cancer is a multifactorial disease, and oxidative stress typically acts alongside other factors such as genetic predisposition, environmental exposures, lifestyle influences, and impaired DNA repair mechanisms. Oxidative stress emerges as a key component of non-thermal effects of RF-EMF exposure. RF-EMF may increase reactive oxygen species (ROS) production and disrupt antioxidant defences, along with elevated oxidative stress biomarkers in the range of ~10–30% under certain exposure conditions (35, 36). In parallel, potential genotoxic effects, including DNA strand breaks and chromosomal instability, have been examined; however, large-scale reviews report inconsistent or weak evidence. RF-EMF lacks sufficient photon energy to directly damage DNA, suggesting that any observed genotoxicity may occur indirectly through oxidative stress pathways (37, 38). These mechanisms have been hypothesized to contribute to carcinogenesis, particularly in tissues with prolonged exposure such as the brain. Nevertheless, epidemiological evidence linking RF-EMF exposure to cancer remains inconclusive, as large cohort studies generally show no consistent increase in cancer incidence, while some case–control studies report elevated risks (OR ≈ 1.5–2.5) in high-exposure groups (39). Overall, although oxidative stress and indirect DNA damage provide biologically plausible pathways, a causal relationship between RF-EMF exposure and cancer development has not been definitively established. Particularly, closer proximity to mobile phone base stations is associated with lower antioxidant levels and higher oxidant markers, supporting a dose-dependent relationship between EMF exposure and oxidative stress. Overall, these findings indicate that RF-EMF–induced oxidative stress may promote a pro-tumorigenic environment by driving cellular damage, metabolic dysregulation, and signaling changes, contributing more to cancer progression than initiation (40).

**Figure 2:**
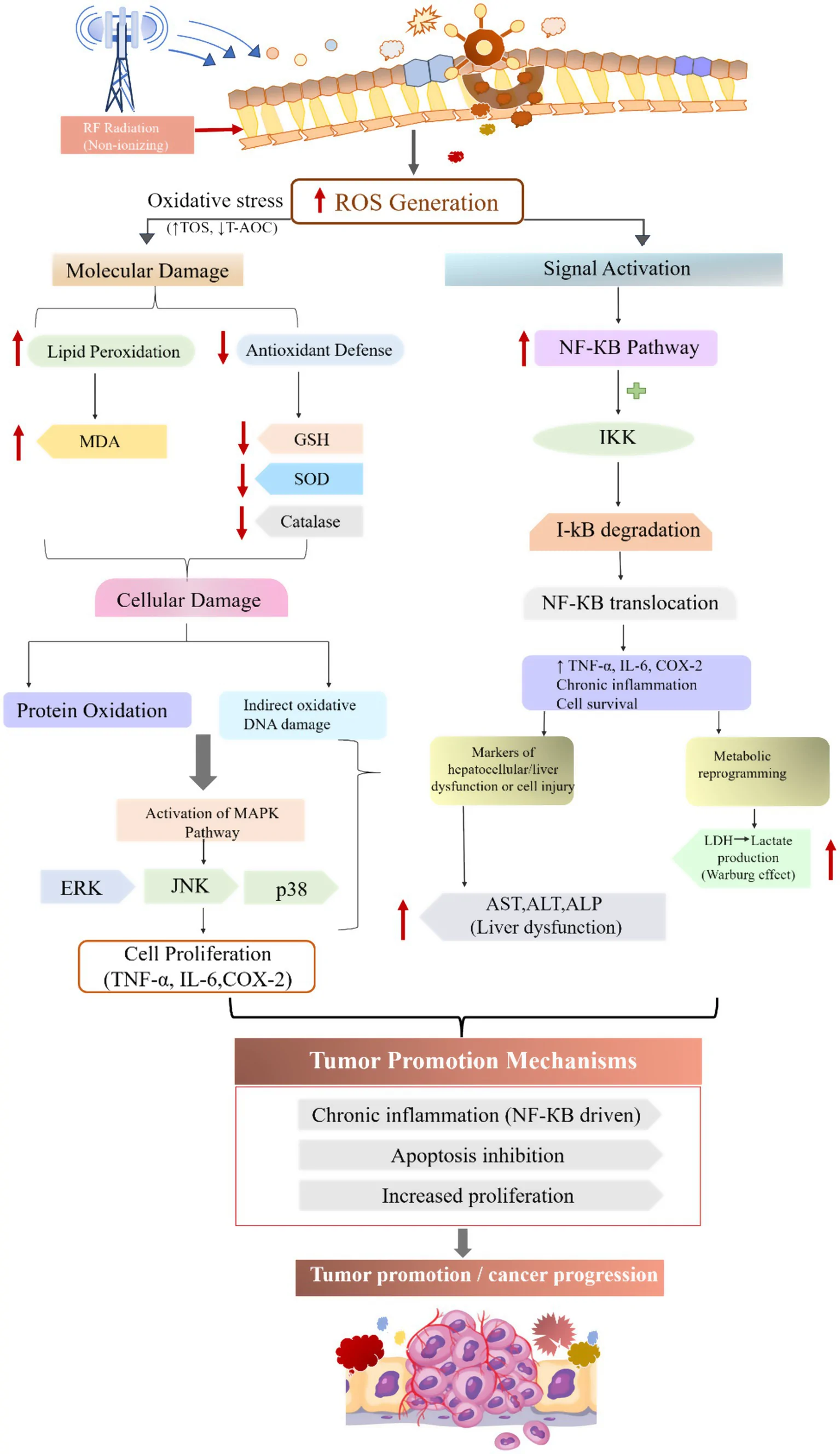
RF-EMF induced ROS generation activates oxidative stress, NF-κB/MAPK signalling, and cellular damage, leading to inflammation and tumor progression: The figure shows how exposure to radiofrequency radiation can contribute to cancer development mainly through oxidative stress. RF radiation increases the production of reactive oxygen species (ROS), which disturbs the normal redox balance of the cell. This leads to lipid peroxidation, reflected by higher levels of malondialdehyde (MDA), and weakens the antioxidant defence system, including glutathione (GSH), superoxide dismutase (SOD), and catalase. As a result, cells experience protein damage and indirect oxidative DNA injury. These changes activate important signalling pathways such as the mitogen-activated protein kinase (MAPK) pathway, involving ERK (extracellular signal-regulated kinase), JNK (c-Jun N-terminal kinase), and p38, which drive cell growth and increase inflammatory molecules like tumor necrosis factor-alpha (TNF-*α*), interleukin-6 (IL-6), and cyclooxygenase-2 (COX-2). At the same time, oxidative stress triggers the nuclear factor kappa B (NF-κB) pathway through IκB kinase (IKK), leading to degradation of IκB and movement of NF-κB into the nucleus, where it promotes chronic inflammation and cell survival. Indicators of cell injury and liver dysfunction, such as lactate dehydrogenase (LDH), aspartate aminotransferase (AST), alanine aminotransferase (ALT), and alkaline phosphatase (ALP), are also elevated, along with increased lactate production linked to the Warburg effect. Altogether, these events support tumor-promoting processes, including persistent inflammation, reduced apoptosis, increased cell proliferation, and metabolic changes marked by higher total oxidant status (TOS) and lower total antioxidant capacity (T-AOC), ultimately may contribute to cancer progression.

The mechanism can be understood with the Figure 2. Although RF-EMF is non-ionizing and does not directly damage DNA, RF-EMF can stimulate intracellular ROS generation, leading to disruption of cellular redox balance (41, 42). This imbalance is characterized by increased total oxidant status (TOS) and decreased total antioxidant capacity (T-AOC), suggesting weakened antioxidant defences (43). Elevated ROS levels cause lipid and protein oxidation and impair antioxidant defenses (glutathione, catalase, superoxide dismutase), resulting in the accumulation of oxidative stress markers such as malondialdehyde (MDA). Oxidative stress may result in hepatocellular injury, reflected by elevated serum levels of liver enzymes (AST, ALT, ALP, and LDH), indicative of cellular damage and altered metabolic function (44, 45). Furthermore, oxidative stress may promote oncogenic signaling and metabolic reprogramming, with increased LDH activity associated with enhanced tumor metabolism, proliferation, and invasiveness (46). Under RF-EMF exposure, increased stress-related mediators and enhanced transamination processes may further aggravate metabolic imbalance and cellular dysfunction (47).

Recent evidence (2015–2025) indicates that mobile phone use is not consistently associated with increased cancer risk, including paediatric brain tumors, glioma, and meningioma. The MOBI-Kids Study by Castaño-Vinyals et al. (48) investigated the association between wireless phone use and neuroepithelial brain tumours in young individuals aged 10–24 years across 14 countries. The study included 899 cases and 1,910 controls and assessed exposure to radiofrequency (RF) and extremely low frequency (ELF) electromagnetic fields. The results showed no increased risk of brain tumours with higher duration or intensity of phone use; in fact, odds ratios tended to decrease with increasing cumulative exposure. These findings were likely influenced by biases such as recall error and changes in phone use due to early symptoms. Overall, the study found no evidence of a causal association, although a small increase in risk cannot be completely ruled out due to residual confounding (48). The MOBI-Kids study, one of the largest international case–control studies on wireless phone use and brain tumors in young people, found no evidence that RF-EMF exposure from wireless phones causes brain tumors. The authors did note, though, that issues like recall bias and other confounding factors mean a small increased risk cannot be completely ruled out. Karipidis et al. (49) also reviewed the biological and health effects of radiofrequency fields above 6 GHz, including those used in 5G networks, at exposure levels below ICNIRP occupational limits. Their review covered 107 experimental studies on topics such as genotoxicity, cell growth, gene expression, cell signaling, and membrane function, as well as 31 epidemiological studies on occupational radar exposure, which served as indirect evidence for 5G frequencies. They concluded that current evidence does not confirm that low-level RF fields above 6 GHz, like those used in 5G, are harmful to human health. However, they pointed out that many studies had limitations, such as poor exposure measurement, lack of temperature control, and not enough independent replication. Some effects observed in studies may be due to heat rather than to RF exposure itself. The authors stressed the need for better-designed experiments with careful exposure assessment and ongoing epidemiological monitoring to track potential long-term health effects of new wireless technologies.

While thermal mechanisms and pathways like oxidative stress and calcium signalling are well-established, recent conceptual perspectives have broadened the discussion of RF-EMF interactions into the broader paradigm of human bioelectromagnetism. Emerging perspectives consider the possibility that endogenous electromagnetic processes associated with cellular membranes, neural activity, cardiac conduction and other physiological functions may interact with external electromagnetic fields via biophysical and biochemical mechanisms. Biophoton signalling has also been proposed as a possible mechanism of intra- and intercellular communication, which in theory could be modified by electromagnetic environments. Recent conceptual work has also proposed tentative quantum-level explanations of interactions between electromagnetic fields and biological systems. However, these mechanisms are hypothesis-generating and not yet shown to be causal pathways between RF-EMF exposure and adverse human health outcomes. Their biological and clinical significance thus needs to be rigorously investigated experimentally using well characterized exposure conditions, quantitative dosimetry, appropriate thermal controls, biologically relevant endpoints, dose–response assessment and independent replication. Combining these emerging mechanistic hypotheses with high-quality epidemiological evidence may help to clarify whether, and under what conditions, RF-EMF exposure could lead to biologically meaningful effects (50, 51) (see Table 2).

**Table 2:** Systematic reviews of epidemiological evidence on RF-EMF exposure and childhood cancer risk.

Author (year)	Type of study	Population type	RF-EMF source type	Distance from the source	Exposure assessment	Type of cancer	Results
Röösli et al. (2019) (120)	Systematic Review	Children (include prenatal exposure)	Mobile phone	NR (varied across included studies)	Self-reported mobile phone use, operator records, and environmental RF-EMF estimates	Brain tumors	No consistent evidence of an association between RF-EMF exposure and childhood brain tumours.
Bodewein et al. (2022) (135)	Systematic review	Children and adolescents	Mobile phone	NR (varied across included studies)	Predominantly questionnaires and interviews; some studies used exposure modelling	Brain tumours and childhood cancers	Evidence was insufficient to establish an association between RF-EMF exposure and childhood cancer because of the limited number and methodological quality of available studies.
Karipidis et al. (2025) (136)	WHO systematic review of observational studies	General population (including children)	Mobile phones and base stations	Near and far-field	Self-reported mobile phone use, environmental exposure modelling, and study-specific exposure assessment	Paediatric brain tumors and leukemia	Moderate-certainty evidence indicated no increased risk of childhood cancer associated with RF-EMF exposure.

NR, Not reported.

Between 2015 and 2025, most case–control studies investigating mobile phone exposure in children and pregnant women have not shown a consistent association with cancer risk. The available evidence is limited and often inconsistent, with no clear exposure–response relationship. Additionally, methodological limitations such as exposure misclassification, recall bias, and inadequate control of confounding factors reduce the reliability of these findings. Therefore, to present the results concisely and to better analyse the underlying reasons, systematic reviews were tabulated instead of individual case–control studies. These studies were selected as they represent evidence generated over the past decade, providing a more comprehensive and updated understanding of the research area.

## 5 Reproductive and developmental health effects

Reproductive and developmental endpoints have been widely investigated due to sensitivity of germ cells and developing organisms to environmental exposures. Some human studies suggest associations between RF-EMF exposure and sperm quality or fetal outcomes. However, the certainty of this evidence remains low, largely due to exposure misclassification and residual confounding. Umbrella reviews and Meta-analysis indicate relatively consistent associations between RF-EMF exposure and reduced sperm motility or viability across experimental and observational studies, with oxidative stress proposed as a plausible biological mechanism. Nonetheless, human observational systematic reviews emphasize very low certainty of effects on sperm count, morphology, and motility underscoring the need for better exposure characterization and well-designed prospective studies (52).

### 5.1 Molecular mechanisms of RF-EMF-induced female reproductive dysfunction

RF-EMF exposure has been associated with potential adverse reproductive effects, including DNA damage in granulosa cells, reduced ovarian follicle numbers, endometrial oxidative stress, and impaired embryonic development in animal models. Studies have shown increased DNA strand breaks in rat granulosa cells following mobile phone radiation higher embryo mortality, growth retardation in exposed chick embryos (53, 54), and reduced follicle counts in offspring of exposed rats (55), while RF-EMF may induce oxidative stress and apoptosis in endometrial tissue and affect fetal cardiac parameters (56, 57). Some studies report no significant impact on embryogenesis or fetal heart rate (58, 59), reflecting inconsistent findings and the need for well-controlled human research.

**Figure 3:**
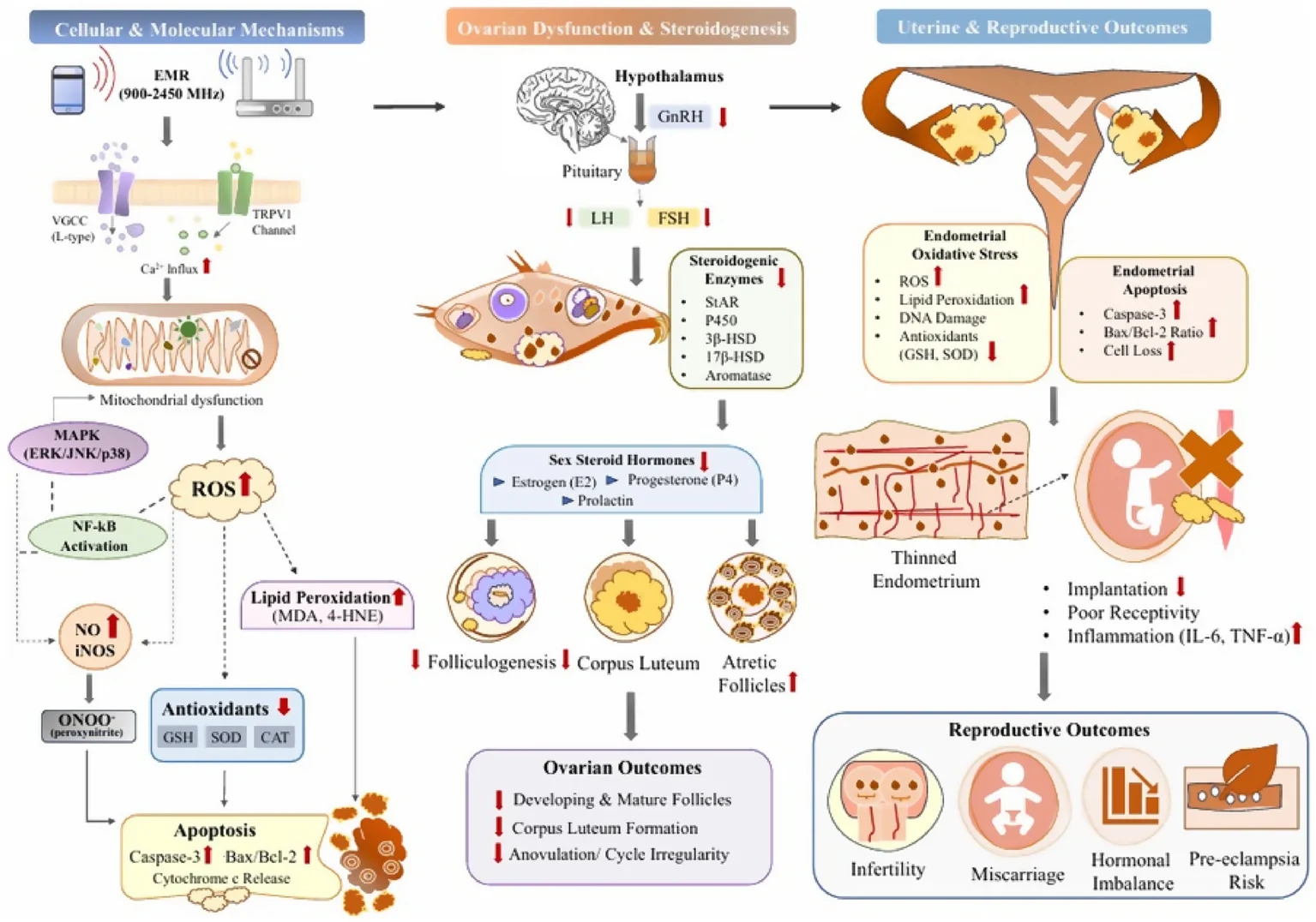
RF-EMF–induced oxidative stress and signalling pathways affecting steroidogenesis and female reproductive function: This figure illustrates the potential mechanisms by which radiofrequency electromagnetic radiation (RF-EMF; 900–2,450 MHz) influences female reproductive health from cellular to systemic levels. At the cellular level, RF-EMF modulates membrane ion channels, including voltage-gated calcium channels (VGCCs) and TRPV1 channels, leading to increased intracellular Ca^2+^ influx and mitochondrial dysfunction. This promotes the generation of reactive oxygen species (ROS) and activates stress signaling pathways such as MAPK (ERK, JNK, p38) and NF-κB. Elevated oxidative stress enhances nitric oxide (NO) and inducible nitric oxide synthase (iNOS) activity, resulting in peroxynitrite formation and subsequent cellular damage. These changes are associated with lipid peroxidation (increased MDA and 4-HNE) and depletion of antioxidant defenses (GSH, SOD, CAT), ultimately triggering apoptosis via caspase-3 activation, altered Bax/Bcl-2 ratio, and cytochrome C release. At the hormonal level, RF-EMF may disrupt the hypothalamic–pituitary–ovarian (HPO) axis, affecting GnRH, LH, and FSH secretion, and downregulating key steroidogenic enzymes (StAR, P450, 3*β*-HSD, 17*β*-HSD, aromatase). This leads to imbalances in estrogen, progesterone, and prolactin, impairing folliculogenesis and ovulation. At the uterine level, increased oxidative stress and inflammation (e.g., IL-6, TNF-α) contribute to endometrial damage, reduced receptivity, and impaired implantation. Collectively, these interconnected mechanisms may contribute to adverse reproductive outcomes, including infertility, hormonal imbalance, and pregnancy complications.

In Figure 3, electromagnetic field exposure from mobile phones leads to reproductive toxicity via oxidative stress-mediated pathways, where excessive ROS generation and depletion of antioxidants such as glutathione and glutathione peroxidase affect cellular homeostasis. EMF may also further increase ROS levels by interacting with the cell membrane and stimulate enzymes like NADH oxidase. This can lead to lipid peroxidation, mitochondrial dysfunction, and reduced cellular detoxification. All these changes result in both structural and functional alterations in reproductive tissues, such as damage to granulosa cells, a decline in ovarian follicle reserve, disruption of folliculogenesis and oogenesis, endometrial cell death, and hormonal imbalances.

It has been reported that chronic exposure to low-intensity GSM-like radiation (900 MHz) in female Sprague–Dawley rats shows mixed outcomes. There have been no pathological changes in Short-term studies, whereas long-term studies indicate significant reduction in survival (~9%). These effects appear to depend on exposure duration and may be influenced by external factors such as birth timing and environmental conditions, suggesting potential cumulative impacts of prolonged RF exposure may also be the contributing factors on which the outcome depends (60).

Mobile phone radiation (1800 MHz) may affect reproduction in mice through oxidative and nitrosative stress, which is characterised by increased ROS, NO, lipid peroxidation, and reduced antioxidant defences. These changes are associated with ovarian degeneration, decreased follicle count, and reduced corpus lutea, along with significant suppression of gonadotropins (LH, FSH), sex steroids (estradiol, progesterone), and steroidogenic proteins (61). Maternal exposure to mobile phone radiation during gestation may affect oogenesis and folliculogenesis in neonatal rats by reducing ovarian hormones (estrogen and progesterone), decreasing primordial follicle numbers, oocyte development, and increasing follicular apoptosis. These effects tend to be stronger exposure during early gestation, suggesting that both timing and duration are important for ovarian development and future fertility (62). In addition, EMF has been associated with serious outcomes such as fetal death, genetic mutations (63), and neurodevelopment disturbances in offspring such as attention deficit disorders (64). At the cellular level, these effects are largely driven by increased oxidative stress, reflected by higher levels of lipid peroxidation markers (e.g., MDA), reduced activity of antioxidant enzyme activity, and activation of apoptotic pathways (caspases, Bax/Bcl-2 imbalance). Despite evidence of structural and functional changes through many studies, the findings are not entirely consistent. Differences in frequency (900–2,450 MHz), exposure duration, and study models limit clear conclusions, highlighting the need for well-standardized exposure conditions and carefully designed studies to better understand EMF-related reproductive effects.

The studies summarized in Table 3, shows mixed and inconsistent findings regarding RF-EMF exposure and health outcomes in pregnant women. Most prospective cohort studies, including those by Baste et al. (65) and Choi et al. (8), did not show any significant association between mobile phone use and adverse pregnancy or neurodevelopmental outcomes. However, some studies suggest that higher level of exposure may pose potential risks. For example, Boileau et al. (66) and Razavimoghadam et al. (67) reported associations between increased mobile phone use and outcomes such as reduced fetal growth, miscarriage, and abnormal birth parameters, although the observed effects were relatively small. Evidence from case–control studies, including Çeleğen et al. (68), also points towards possible associations with specific congenital anomalies, while Bektaş et al. (69) reported changes in oxidative stress markers, indicating possible biological effects. Overall, the current evidence does not support a consistent association, but it also does not completely rule out potential risks, particularly at higher exposure levels or under specific conditions (Table 3). Current evidence on prenatal exposure to mobile phone use and electromagnetic fields shows inconsistent and limited associations with pregnancy and early life outcomes. Most studies report increased risks such as reduced fetal growth, congenital abnormalities, miscarriage and behavioral problems. These findings are mostly observed at higher exposure level and are not consistent across studies. In many cases, no significant associations are found at lower exposures. There is also evidence suggesting interaction effects with other environmental factors (e.g., lead exposure) and possible biological changes like oxidative stress, though their clinical importance is unclear. Overall, there is no strong or consistent causal evidence, and observed effects are likely influenced by confounding factors.

**Table 3:** Primary human epidemiological studies of RF-EMF exposure and pregnancy/developmental outcomes.

Sr. no.	Author (year)	Type of study	Population type	RF-EMF source type	Distance from source	Exposure assessment	Outcome	Risk estimate	Results
1.	Baste et al. (2015) (65)	Prospective cohort study	Pregnant women (MoBa cohort, Norway) and their children	Mobile phones (maternal and paternal use)	Not explicitly specified (typical use near head/body)	Self-reported mobile phone use (frequency categories: none, low, medium, high)	Pregnancy outcomes: low birth weight, preterm birth, small for gestational age	No significant increased risk (ORs ~ 1, not statistically significant)	No consistent association between parental mobile phone use and adverse pregnancy outcomes.
2.	Boileau et al. (2020) (66)	Prospective cohort study	Pregnant women and newborns	Maternal mobile phone use during pregnancy	Not directly measured; presumed close to abdomen/body during routine use	Self-reported questionnaire on mobile phone use during pregnancy (frequency/duration categories)	Fetal growth indicators: birth weight, birth length, head circumference, fetal growth restriction	>30 min/day vs. < 5 min/day: aOR = 1.54 (95% CI: 1.03–2.31), *p* = 0.037; 5–15 min: aOR = 0.98 (0.58–1.65); 15–30 min: aOR = 1.68 (0.99–2.82, NS)	High mobile phone use (>30 min/day) was significantly associated with increased risk of reduced fetal growth (AUDIPOG ≤10th percentile). Lower exposure categories showed no significant association.
3.	Razavimoghadam et al. (2025) (67)	Prospective cohort study	Pregnant women in Yazd, Iran and their newborns	Cell phones, cordless phones, Wi-Fi (RF-EMF sources)	Not directly measured; inferred from usage patterns and proximity to devices	Structured questionnaire assessing frequency/duration of exposure to common EMF sources during pregnancy	Miscarriage, preterm labor, abnormal birth weight, abnormal height, abnormal head circumference	Miscarriage: RR = 1.0061 (95% CI: 1.003–1.0093), *p* < 0.001; Abnormal birth weight: RR = 1.0012 (1.0004–1.002), *p* = 0.002; Abnormal height: RR = 1.0014 (1.0004–1.0023), *p* = 0.003	Longer duration of mobile phone calls during pregnancy was significantly associated with increased risk of miscarriage, abnormal birth weight, and abnormal height. Cordless phone use also increased risk of abnormal birth weight.
4.	Çeleğen and Özgül (2024) (68)	Case–control study	Infants <2 years (57 CAKUT cases, 57 healthy controls) and their mothers	Maternal mobile phone use during pregnancy (RF-EMF)	Partially assessed (proximity when not in use; not significant)	Structured questionnaire on mobile phone use (frequency, duration, daily use habits during pregnancy)	Congenital anomalies of kidney and urinary tract (CAKUT)	Longer call duration: *p* = 0.001; Higher effective SAR: *p* = 0.03; Proximity (not in use): NS	Longer maternal call duration and higher effective SAR exposure were significantly associated with increased risk of CAKUT. Phone proximity when not in use was not associated.
5.	Choi et al. (2017) (8)	Prospective cohort study	Pregnant women and their children (birth cohort followed up to 3 years, South Korea)	Maternal mobile phone use (RF-EMF); environmental RF radiation; co-exposure to lead	Not directly measured; inferred from phone use patterns and environmental exposure	Maternal questionnaire on mobile phone use (frequency/duration); RF exposure modeling; blood lead levels measured	Neurodevelopment (MDI and PDI scores at 6, 12, 24, 36 months; Bayley Scales)	No significant association for RF-EMF overall; interaction effect: increased risk of low PDI with higher call time in high lead group (p-trend = 0.008); decreasing MDI with increasing call time/frequency (p-trend = 0.05 and 0.007)	No independent association between prenatal RF-EMF exposure and neurodevelopment. However, combined exposure with high prenatal lead levels showed significant adverse effects, suggesting interaction/modification effect.
6.	Bektaş et al. (2018) (69)	Observational (cross-sectional)	Pregnant women and their newborns (cord blood samples at delivery)	Maternal mobile phone use during pregnancy (RF-EMF)	Not directly measured; assumed close proximity to body during routine phone use	Self-reported mobile phone use (grouped by usage duration, e.g., low vs. high users)	Biochemical parameters in cord blood (e.g., oxidative stress markers, antioxidant enzyme levels)	Significant differences observed in some biochemical markers between exposure groups	Higher maternal mobile phone use was associated with alterations in cord blood biochemical parameters, particularly indicators of oxidative stress and antioxidant status. Findings suggest possible biological effects, but clinical significance remains unclear.

## 6 Neurophysiological effects and brain function

RF-EMF exposure has also been linked to potential neurophysiological effects, including alterations in brain electrical activity, sleep patterns, and cognitive function. Experimental studies using electroencephalography (EEG) have reported changes in brain wave activity following acute exposure, particularly in frequency bands associated with sleep regulation. However, epidemiological evidence remains inconsistent, and many observed effects are subtle and within normal physiological variability. Recent human studies (2023–2024) emphasize that while short-term neurophysiological responses may occur, there is insufficient evidence to establish clinically significant long-term neurological effects.

### 6.1 Mechanistic pathway underlying neurological effects

RF-EMF influences the central nervous system through both thermal and non-thermal effects, which leads to changes in brain function and behaviour. It has found to be associated with alterations in neurotransmitters signalling, EEG patterns, and cerebral blood flow, which may interfere with learning, memory, and neurobehavioral patterns (70, 71). Prolonged exposure may lead to oxidative stress, neuronal damage, and apoptosis; it also causes structural changes in brain regions such as the cortex and hippocampus (72, 73). Electromagnetic radiation significantly disrupts multiple neurotransmitter systems in the brain, along with biogenic amines, amino acid neurotransmitters, and associated signalling mechanisms, which results in affecting cognition, behaviour, and neuronal homeostasis. Among biogenic amines, dopamine levels along with its metabolite DOPAC (3,4-dihydroxyphenylacetic acid) are consistently found to be reduced following RF-EMF exposure (900–1800 MHz) in regions such as the hippocampus and striatum, leading to impaired learning and memory. High intensity exposure suppresses dopamine, and very high-power densities may increase its turnover without interfering with total levels. Prenatal exposure demonstrates a dose-dependent effect, low exposure increases dopamine and higher exposure decreases it, indicating developmental neurotoxicity (74–76). Norepinephrine and epinephrine levels are altered, with prolonged RF-EMF exposure reducing their concentration in the hippocampus region, while low-intensity exposure may increase norepinephrine, this reflects a biphasic response that is also observed in prenatal conditions (76, 77). Serotonin (5-HT) metabolism is also affected, primarily through increased turnover and elevated levels of its metabolite 5-HIAA, and in some cases increased serotonin levels in brain regions associated with memory impairment, although findings remain inconsistent in prenatal studies (74, 76, 78). In addition to monoamines, RF-EMF disrupts amino acid neurotransmitters, altering glutamate and aspartate levels. Short-term exposure typically reduces their concentration, whereas long-term exposure shows variable effects depending on intensity, often impairing memory and learning. It may also modify NMDA receptor subunits (NR2A and NR2B), which results in affecting synaptic transmission (78–81).

**Figure 4:**
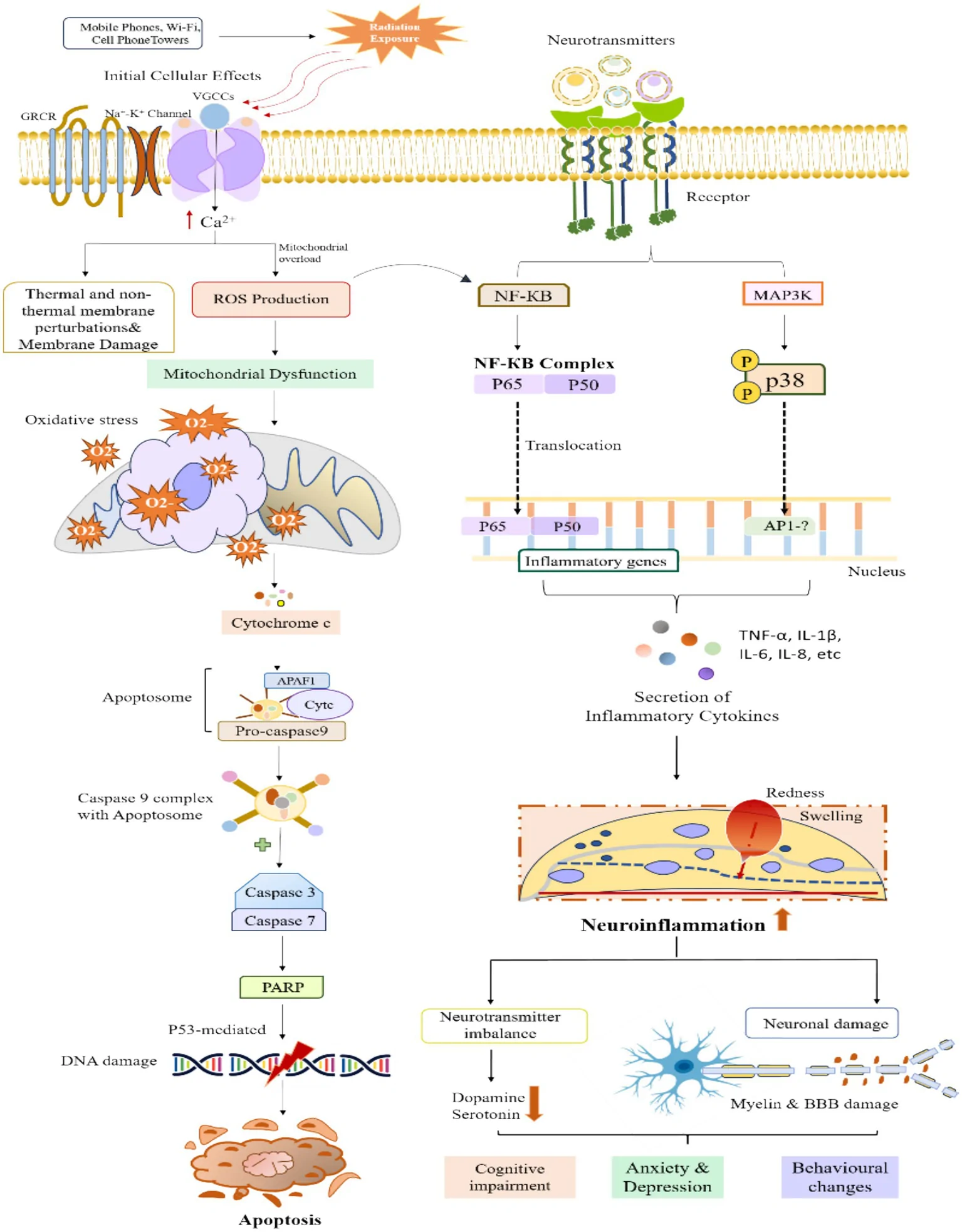
RF-EMF induces oxidative stress, mitochondrial dysfunction, inflammatory signalling, and apoptosis which leads to neuronal damage and associated cognitive and behavioural impairments: The figure shows how electromagnetic radiation (RF-EMF) from sources like mobile phones, Wi-Fi, and cell towers affects cells and brain function. RF-EMF causes thermal and non-thermal membrane disturbances, leading to increased production of reactive oxygen species (ROS) and resulting in mitochondrial dysfunction with excess superoxide (O_2_^−^) generation. This triggers the release of cytochrome c (Cyt c), which binds with apoptotic protease activating factor-1 (APAF1) to activate pro-caspase-9, followed by activation of caspase-3 and caspase-7, leading to apoptosis and damage to poly (ADP-ribose) polymerase (PARP) and DNA. Simultaneously, oxidative stress activates inflammatory signaling pathways such as nuclear factor kappa B (NF-κB), including its subunits p65 (RelA) and p50, and the mitogen-activated protein kinase (MAP3K) pathway, which activates p38 mitogen-activated protein kinase (p38 MAPK), along with transcription factors like activator protein-1 (AP-1), promoting expression of inflammatory genes. These combined effects lead to cellular swelling, neuronal damage, and disruption of neurotransmitter balance, ultimately resulting in cognitive impairment, anxiety and depression, and behavioural changes.

Inhibitory neurotransmission is also affected, with GABA levels and release generally reduced, however prolonged exposure may increase GABA and glycine levels in some cases, disrupting the glutamate-GABA balance and receptor expression (82, 83). Acetylcholine, a key neurotransmitter involved in memory processes, shows mixed responses with short-term increases but reduced cholinergic activity upon prolonged exposure, accompanied by change in AChE and ChAT enzyme activity and receptor expression (84–87). As discussed in Figure 4, RF-EMF also affects peptide neurotransmitters such as opioid peptides and increases nitric oxide (NO) levels, causing neuronal dysfunction and neurotoxicity (88, 89). These neurochemical alterations are driven by interconnected mechanisms, including electrophysiological changes such as increased cortical excitability and altered EEG patterns (reduced alpha and increased beta/gamma activity), along with disturbances in sleep and reaction time (90–94). At the cellular level, RF-EMF disrupts membrane permeability and calcium homeostasis, a critical factor in neurotransmitter release and synaptic signalling, leading to abnormal neuronal activity or damage. It induces oxidative stress through increased generation of ROS, inflammatory pathways activation, and neuronal apoptosis. Together, these effects disturb neuronal signalling, and contribute to cognitive dysfunction.

Some studies have also linked RF-EMF exposure to sleep disturbances, mood disorders, and reduced cognitive performance, although the findings remain inconsistent (95, 96), while RF-EMF can significantly affect neurophysiological processes at cellular and molecular levels, current evidence does not strongly support a direct causal link with brain tumors. Overall, RF-EMF exposure may affect brain health by disrupting normal neuronal activity and signalling.

In total 17 studies were tabulated assessing RF-EMF exposure and neurocognitive, behavioural, and sleep outcomes in children and adolescents show inconsistent and heterogeneous findings (Table 4). Several cohort studies, including Brzozek (97), Bhatt (98), and Roser (99), reported no consistent association between RF-EMF exposure and cognitive or behavioural outcomes. Similarly, Choi et al. (8) found no independent effect of RF-EMF on neurodevelopment, with adverse outcomes observed only in the presence of co-exposure to lead. However, some studies suggested possible associations, particularly with higher exposure or behavioural factors. Foerster (100) reported reduced figural memory with higher RF-EMF dose, while Cabré-Riera et al. (101, 102) found associations with lower non-verbal intelligence and shorter sleep duration, respectively. Studies such as Sudan et al. (103) and Birks et al. (104) reported increased risks of behavioral problems with prenatal and postnatal mobile phone exposure, although residual confounding is likely. Cross-sectional studies, including Durusoy et al. (105), Chiu et al. (106), and Zheng et al. (107), reported associations with symptoms such as headache, fatigue, and sleep disturbances; however, these findings are limited by self-reported exposure and potential bias. Overall, the evidence suggests that observed associations are more likely related to behavioural and lifestyle factors rather than direct RF-EMF exposure, with no consistent causal relationship established (Table 4).

**Table 4:** Epidemiological evidence on neurological effects of RF-EMF exposure in children.

Sr. no.	Author (year)	Type of study	Population type	RF-EMF source type	Distance from source	Exposure assessment	Outcome	Statistical test	Risk estimate	Results
1.	Cabré-Riera et al. (2022) (102)	Cross-sectional	Preadolescents (9–12 yrs., *n* = 1842)	Mobile phone, DECT, WiFi, base stations	Mixed (near + far field)	Integrated RF-EMF dose model (SAR-based, geospatial + personal measurements)	Sleep (objective + subjective)	Linear regression	*β* = −11.9 min sleep (CI − 21.2 to −2.5)	High evening RF dose linked to shorter sleep duration; likely behavioral confounding
2.	Çöl et al. (2021) (133)	Cohort	Mother–child pairs (1 mo–5 yrs., *n* = 400)	Mobile phone, WiFi (prenatal exposure)	Near-field + environmental	Questionnaire (parent-reported prenatal exposure)	Sleep disturbances	Logistic regression	OR 7.48 (night waking), OR 9.89 (crying), OR 4.95 (sleep onset issues)	Strong associations between prenatal exposure and sleep problems
3.	Cabré-Riera et al. (2021) (101)	Cross-sectional	Preadolescents and adolescents (*n* = 3,213)	Mobile phone, DECT, base stations	Mixed	Integrated RF dose model (SAR + geospatial + personal data)	Cognitive function (intelligence)	Linear regression	*β* = −0.10 per 100 mJ/kg/day	Higher overall estimated whole-brain RF-EMF doses from all RF-EMF sources together and from phone calls were associated with lower non-verbal intelligence score
4.	Brzozek et al. (2019) (97)	Cohort	Children (9–11 yrs., *n* = 619)	Mobile phone	Near-field	Questionnaire + Monte Carlo simulation	Cognitive function	Linear regression	*β* = −0.023 to −0.024 (RT), *β* = 4.53 (accuracy)	Small improvements; overall near-null findings
5.	Foerster et al. (2018) (100)	Cohort	Adolescents (12–17 yrs., *n* = 895)	Mobile and cordless phone	Near-field (brain exposure)	Operator data + questionnaire + SAR modelling	Memory (figural)	Linear regression	*β* = 0.22 (95% CI: −0.47, 0.03); in the full sample (*n* = 669) and 0.26 (in the operator sample)	Significant reduction in figural memory with higher RF dose
6.	Bhatt et al. (2017) (98)	Cohort	Children (9–11 yrs., *n* = 619)	Mobile and cordless phone	Near-field	Questionnaire (calls/week, change over time)	Cognitive function	Linear regression	*β* = −0.030 (response inhibition), *β* = 6.22 (executive function)	Higher cordless phone use than mobile phones; no consistent associations between changes in phone use (MP/CP) and cognitive function in children
7.	Schoeni et al. (2017) (137)	Cohort	Adolescents (*n* = 439)	Mobile phone, cordless phone	Near-field + cumulative dose	Operator data + questionnaire + SAR modeling	Symptoms	Logistic regression	OR for tiredness: 2.33; 95%CI: 1.54 to 3.52 for cross-sectional analyses and OR: 2.70; 95%CI:1.52 to 4.80 for cohort analyses	Health symptoms in adolescents were more strongly associated with wireless device use behaviours (e.g., texting, data use) than with RF-EMF exposure, suggesting no causal link between RF-EMF dose and reported symptoms
8.	Papadopoulou et al. (2017) (138)	Cohort	Children (3–5 yrs., *n* = 45,389/17,310)	Mobile phone (prenatal)	Near-field	Maternal questionnaire	Language, motor, communication	Logistic regression	OR 0.83 (language), OR 0.82 (motor)	Improved developmental outcomes; no adverse effects observed
9.	Durusoy et al. (2017) (105)	Cross-sectional	Adolescents (~15.6 yrs., *n* = 2,150)	Mobile phone, base stations	Near + far field	Questionnaire + environmental measurements + SAR data	Multiple symptoms	Logistic regression	OR 1.90 (headache), OR 1.78 (fatigue), OR 1.53 (sleep issues)	Multiple significant symptom associations with mobile phone use
10.	Choi et al. (2017) (8)	Cohort	Infants (6–36 months, *n* = 1,198)	Mobile phone (prenatal), environmental RF-EMF	Near-field + far-field	Maternal questionnaire + personal exposimeter + blood lead measurement	Neurodevelopment (mental, psychomotor)	Trend analysis / regression	*p*-trend = 0.007 (mental), 0.008 (psychomotor)	Decreased development only in high lead exposure group; no independent RF-EMF effect
11.	Birks et al. (2017) (9)	Multinational cohort analysis	Children (5–7 yrs., *n* = 83,884)	Mobile phone (prenatal)	Near-field	Maternal questionnaire (calls/day during pregnancy)	Behavioural problems (hyperactivity, emotional)	Logistic regression	OR 1.11 (2–3 calls/day), OR 1.28 (≥4 calls/day)	Increased hyperactivity/inattention with higher prenatal phone use; confounding likely
12.	Sudan et al. (2016) (103)	Cohort	Children (7–11 yrs., *n* = 47,721)	Mobile phone (prenatal/postnatal)	Near-field	Maternal questionnaire	Emotional and behavioural problems	Logistic regression	OR 1.58 (combined exposure), OR 1.41 (prenatal)	Consistent association across time points, both prenatal and postnatal exposures may be associated with increased risks of emotional and behavioural difficulties in children.
13.	Roser et al. (2016) (99)	Cohort	Adolescents (12–17 yrs., *n* = 439)	Mobile phone, base stations	Mixed	Dosimetry + operator + modelling	Concentration and behavioural problems	Linear regression	*β* = −3.50 to −6.00 (cross-sectional) *β* = 0.98 (cross-sectional), *β* = −0.61 (longitudinal)	No evidence of an association between RF-EMF exposure and behaviour or concentration capacity in adolescents
14.	Schoeni et al. (2016) (139)	Cohort	Adolescents (12–17 yrs., *n* = 439)	Base stations, broadcast transmitters	Far-field	Geospatial RF modelling	Symptoms	Logistic regression	OR for tiredness (2.94, 95%CI: 1.43 to 6.05)	Inconsistent findings with self-reported well-being symptoms in adolescents
15.	Chiu et al. (2015) (106)	Cross-sectional	Children (11–15 yrs., *n* = 2042)	Mobile phone	Near-field	Questionnaire (CAPI, parent-assisted)	Headache, migraine, skin symptoms	Logistic regression	OR 1.42 (headache), OR 1.84 (skin itching)	Significant association between phone use and symptoms
16.	Huss et al. (2015) (115)	Cohort	Children (5–8 yrs., *n* = 2,361)	Mobile phone, WiFi, base stations	Mixed	Questionnaire + modelled exposure	Sleep outcomes	Poisson regression (IRR)	IRR 1.43 (sleep duration), IRR 1.51 (night waking)	Associations likely due to behavioural factors, not RF exposure
17.	Zheng et al. (2015) (107)	Cross-sectional	Children (9–12 yrs., *n* = 746)	Mobile phone	Near-field	Questionnaire	Fatigue	Logistic regression	MP usage (OR 1.85; 95% CI 1.07 to 3.22); the daily duration of MP calls (OR 2.98; 95% CI 1.46 to 6.12);	Significant association between use and fatigue

## 7 Biological and physiological markers

Biological and physiological markers provide important insight into potential mechanisms underlying RF-EMF-related health effects, particularly in the context of non-thermal interactions. Among these, oxidative stress markers are the most consistently reported, with several studies demonstrating increased levels of reactive oxygen species (ROS), malondialdehyde (MDA), and 8-hydroxy-2′-deoxyguanosine (8-OHdG), alongside reductions in antioxidant enzymes such as superoxide dismutase (SOD) and glutathione peroxidase (GPx), typically in the range of 10–30% (108, 109). Genotoxicity markers, including DNA strand breaks and micronucleus formation, have also been investigated, although findings remain inconsistent and are thought to occur indirectly via oxidative stress rather than direct DNA interaction (110). Hormonal markers, particularly melatonin and cortisol, suggest possible disruption of circadian rhythms, with some studies reporting reductions in melatonin levels of approximately 10–20%, though results are variable (111). Neurological markers, such as electroencephalographic (EEG) activity and neurotransmitter levels, indicate subtle neurophysiological changes without clear clinical implications. Cardiovascular markers, including heart rate variability, show minor alterations but no consistent adverse effects, while reproductive markers especially sperm motility and quality suggest potential sensitivity to RF-EMF exposure, although evidence remains inconclusive (52). Although these biomarkers suggest potential mechanistic plausibility, their direct translation into adverse health outcomes has yet to be established.

## 8 Discussion

A wide range of epidemiological and experimental research from Europe, Asia, and the Middle East has examined the potential health effects of RF-EMF exposure from mobile phones and towers among children and pregnant woman but as subgroups. In these studies, sample sizes varied substantially, ranging from 18 participants in experimental setups to over 3,000 in large population-based cohort studies (112–114). Most studies focused on school-aged children and preadolescents (8–17 years), with exposure durations ranging from short-term laboratory sessions (5–50 min) to daily phone use or residential exposure assessed over several years (Table 4). Exposure sources and assessment methods differed significantly across studies. Experimental studies typically applied GSM signals at 902–925 MHz to stimulate direct near-field exposure from mobile handsets, while larger observational and cohort studies measured environmental or personal RF exposure from multiple sources, including mobile base stations, Wi-Fi routers, cordless phones, and handheld devices. Self-reported daily mobile use ranged from a few minutes to several hours, with some cohorts (106, 115) categorising usage into detailed tiers (e.g., 20 min/day, 21–40 min/day, and 60 min/day, or 1–3 calls per week to 3 calls per week). In older adolescent populations, regular mobile phone use extended beyond two years in several longitudinal studies (98, 116).

The existing evidence is constrained by heterogeneity in exposure assessment, study design and duration, hindering consistent conclusions. Most studies rely on short-term or self-reported exposure data, which reduces reliability, particularly in evaluating long-term health effects. Consequently, there remains a clear need for well-designed, standardized longitudinal studies to better understand RF-EMF exposure impacts in vulnerable populations. Additionally, careful consideration of methodological limitations are needed to interpret the available evidence. Many epidemiological studies used self-reported mobile phone use or surrogate measures such as residential distance to base stations, which may lead to exposure misclassification and recall bias. Furthermore, confounding factors like socioeconomic status, screen time, lifestyle, environmental co-exposures and access to health care cannot be fully ruled out. Thus, statistically significant associations reported in some studies should be treated with caution, particularly when the effect sizes are small, and inconsistent across studies or lack biological plausibility.

Although the epidemiological evidence remains limited, experimental studies have reported biological effects of RF-EMF exposure, including oxidative stress, altered calcium signaling, mitochondrial dysfunction, DNA damage, neuroinflammation, and changes in neurodevelopment and reproductive function under certain exposure conditions (117–119). These mechanistic findings appear to contrast with the largely inconclusive evidence from human observational studies. This discrepancy may reflect differences in exposure characteristics, controlled experimental conditions, species-specific responses, and the inherent limitations of epidemiological research in accurately quantifying long-term RF-EMF exposure. Therefore, integrating mechanistic, experimental, and high-quality epidemiological evidence will be essential to clarify the potential health effects of RF-EMF exposure and improve future risk assessment. Emerging conceptual frameworks of human bioelectromagnetism, including proposed roles for endogenous electromagnetic processes and biophoton signalling, provide additional hypotheses for investigating electromagnetic interactions in biological systems; however, these concepts remain hypothesis-generating and insufficiently validated for causal risk assessment (51, 120).

Cancer was included as one of the key outcomes in this review. While the review by Ramírez-Vázquez et al. (121) did not find a consistent association between RF-EMF exposure and cancer incidence, experimental evidence has reported biological effects such as increased oxidative stress, including a 10–30% rise in 8-hydroxy-2′-deoxyguanosine (8-OHdG) levels. These findings suggest that, although epidemiological evidence remains inconclusive, underlying mechanistic pathways cannot be entirely disregarded (121). In contrast, studies identified increased odds of glioma (OR ≈ 1.8–2.5) among long-term users (>20 years), particularly with ipsilateral exposure, although these findings remain debated due to potential recall bias and inconsistency with cohort data (122). It is very difficult to conclude whether cancer can be an outcome of RF-EMF radiation or not. In children, previous investigations have explored environmental RF-EMF exposure (e.g., from fixed-site transmitters such as base stations) in relation to childhood leukemia risk (17). However, the overall evidence remains inconclusive with no consistent association, largely due to heterogeneity in exposure assessment methods and potential bias across studies. Prenatal exposure (maternal exposure during pregnancy) is often only indirectly considered, and there is notable lack of well-designed cohort or case–control studies specifically evaluate in utero RF-EMF exposure and subsequent cancer risk in offspring. Moreover, many studies rely on proxy measures such as distance from transmitters or self-reported mobile phone use, leading to exposure misclassification and uncertainty in risk estimation. Overall, the review concludes that current epidemiological evidence is insufficient to establish a causal relationship between RF-EMF exposure and cancer outcomes in children or during pregnancy, and strongly recommends well-designed prospective studies with accurate, individual-level exposure assessment, particularly focusing on vulnerable populations such as foetuses and children.

For cognitive and behavioural outcomes, modest associations have been reported, particularly in children and adolescents. Studies with improved exposure assessment (123), indicate small effect sizes (OR ≈ 1.3–1.6) for outcomes such as sleep disturbances and attention-related changes; however, these associations are frequently attenuated after adjustment for confounding factors, including screen time, lifestyle patterns, and psychosocial influences. In contrast, evidence for reproductive and developmental outcomes in pregnant women remains largely inconsistent, with most studies reporting no robust or clinically significant effects. From a mechanistic perspective, experimental research, including work by Senturk et al. (124) suggests biological plausibility through pathways such as increased oxidative stress (approximately 10–30%) and potential indirect DNA damage. Nevertheless, these mechanistic findings have not been consistently reflected in epidemiological data, and a causal relationship with adverse reproductive or cognitive outcomes remains unestablished. This gap between experimental and population-based evidence continues to complicate interpretation.

Evidence specific to pregnancy and birth outcomes in humans remain limited, with few high quality epidemiological studies published in last decade and comprehensive systematic synthesis still in progress. Experimental mammalian and *in vitro* studies provide supportive biological context but have uncertain translational relevance to typical environmental or personal use exposure levels (125, 126). WHO commissioned reviews similarly conclude that human observational studies show little to no consistent effects of mobile phone use on key sperm parameters. Although, overall evidence is very low due to methodological limitations and poor exposure characterization (52). However, evidence indicating clinically relevant effects at typical environmental exposure levels remains limited. In pregnant populations, potential neurological concerns related to RF-EMF exposure are addressed indirectly through neurodevelopment and cognitive outcomes in offspring rather than direct assessment of maternal brain function (16). Current epidemiological evidence does not demonstrate associations between maternal RF-EMF exposures with adverse neurodevelopment outcomes in children (127). Nevertheless, interpretation of these findings is limited by the small number of long term prospective birth cohorts, reliance on self-reported exposure metrics and imprecise exposure characterization, which collectively reduce confidence in causal inference. Importantly, this apparent lack of conclusive evidence should not be interpreted as evidence of the absence of risk. Rather, it highlights a significant scientific and public health research gap, given that these populations are considered biologically vulnerable and are increasingly exposed to RF-EMF through the widespread use of wireless technologies from early life. The limited certainty of the available evidence largely reflects methodological challenges, including heterogeneous exposure assessment methods, reliance on self-reported mobile phone use, variations in outcome definitions, inadequate control of confounding factors, and a lack of well-designed prospective longitudinal studies. Consequently, the current evidence base remains insufficient to draw definitive conclusions regarding long-term health effects.

Most studies have focused only on outcomes and have not adequately addressed the underlying mechanisms, even though mechanisms play a very important role in cancer development. Cancer-related biomarkers should also be evaluated and incorporated into the results. In reproductive outcomes, although some factors are considered, not all relevant parameters are included for example; androgen and testosterone levels are often overlooked. Very few studies comprehensively assess all hormones before drawing conclusions. Similarly, neurological disorders are often merely reported, without identifying their underlying causes. Future studies building on this review should take these aspects into careful consideration. The overall balance of evidence across health outcomes remains mixed. No consistent association between RF-EMF exposure and cancer risk was reported in all three systematic reviews or large epidemiological investigations included in this review. Regarding reproductive health, about half of the epidemiological studies found no significant associations, while the others reported weak associations with some adverse outcomes, often with methodological limitations. Several studies reported statistically significant associations between sleep disturbances, cognitive performance, headaches or behavioural symptoms and neurological outcomes. However, a considerable number of well-designed cohort studies reported null or near-null findings. Importantly, many of the positive associations were based on self-reported exposure, cross-sectional designs or proxy exposure measures and therefore remain susceptible to recall bias, exposure misclassification and residual confounding. Statistical significance should not be taken as evidence of clinical or biological significance without considering effect size, consistency across studies, quality of exposure assessment, and biological plausibility.

### 8.1 Practical precautionary measures

Although current epidemiological evidence does not establish a causal relationship between RF-EMF exposure and adverse health outcomes, simple precautionary measures may help reduce unnecessary exposure, particularly among children and pregnant women while scientific uncertainties remain (128). These measures include limiting the duration of mobile phone calls, using speaker mode or wireless hands free devices during prolonged calls, preferring text messaging where practical, avoiding prolonged direct body contact with active mobile phones, particularly during pregnancy, and encouraging age appropriate mobile phone use among children (129–132). These measures are precautionary, low cost, and consistent with recommendations from public health authorities and should not be interpreted as evidence that RF-EMF exposure below current international guideline limits causes adverse health effects (133, 134). The effectiveness of commercially available EMF shielding or protective accessories remains insufficiently validated and therefore cannot currently be recommended as evidence based exposure reduction strategies.

## 9 Future research directions

The limitations identified across the epidemiological and experimental literature indicate several priorities for future research.

### 9.1 Longitudinal pregnancy and developmental cohorts

Large prospective birth cohorts should start exposure assessment in pregnancy and follow up to childhood and adolescence. Repeated RF-EMF measurements should be combined with validated individual exposure models, personal dosimetry, mobile device use, residential measurements and information on wireless infrastructure. Longitudinal follow-up should assess developmental, cognitive, behavioural, sleep, neurological, reproductive and cardiometabolic outcomes at multiple time points. These designs would lessen reliance on retrospective exposure assessment and improve assessment of possible exposure-response relationships across critical developmental windows.

### 9.2 Real-world and multi-source exposure assessment

Future studies should go beyond single device or single source exposure estimates, and characterizes the complex RF-EMF environment experienced in daily life. This should include mobile phones, Wi-Fi routers, wearable devices, Bluetooth-enabled equipment, connected household technologies, base stations and other wireless Internet-of-Things devices. Short-term and cumulative exposure patterns should be estimated by combining personal exposimetry, geospatial modelling, operator recorded device activity, repeated measurements and time-resolved exposure assessment. Importantly, cumulative exposure should be interpreted as repeated or time-integrated exposure rather than biological accumulation of RF-EMF in tissues. Studies should address whether simultaneous exposure from multiple RF-emitting sources modifies exposure-response relationships or introduces additional uncertainty into standard single-source models.

### 9.3 RF-EMF and chemical or environmental co-exposures

Future studies should explore whether RF-EMF exposure interacts with other environmental stressors such as heavy metals, air pollution, pesticides, endocrine-disrupting chemicals, environmental noise and psychosocial stress. Studies should employ formal interaction models, not assume potentiation from co-exposure. Special attention should be paid to pregnancy where fetal development takes place in the context of multiple simultaneous environmental exposures. Thus, the potential interaction between RF-EMF and chemical pollutants should be studied using exposome-based approaches and mechanistically informed experimental designs.

### 9.4 Mechanistic integration and biomarkers

Future research should evaluate exposure, intermediate biological responses, and clinically relevant outcomes simultaneously. Mechanistic endpoints may include oxidative stress, inflammatory signaling, mitochondrial function, DNA integrity, endocrine parameters, neurophysiological measures, and other validated biomarkers. Comprehensive assessment of relevant reproductive hormones and functional outcomes should be included in reproductive studies, instead of isolated biomarkers. Likewise, neurological studies should include objective neurocognitive, sleep, electrophysiological, and neuroimaging endpoints, as appropriate.

### 9.5 Research independence, transparency, and regulatory review

Future research should be as transparent as possible in its funding, conflicts of interest, exposure protocols, analytical choices and data availability because the assessment of RF-EMF involves fast-moving technologies and large commercial stakes. We should encourage independent replication and collaborative studies involving exposure scientists, epidemiologists, clinicians, engineers, toxicologists, and regulatory scientists. As wireless technologies, exposure patterns, dosimetry methods and biological evidence evolve, international scientific and public-health organizations should periodically review the evidence. Any amendments to exposure advice should be evidence based, and proportionate to the certainty of available evidence.

Together these priorities would help to close the current gap between experimental and epidemiological evidence and contribute to more reliable risk assessment for pregnant women, fetuses, children and adolescents.

## 10 Conclusion

The current evidence indicates that the association between RF-EMF exposure and health outcomes in vulnerable groups particularly children and pregnant women remains uncertain and not yet definitively resolved. Although mechanistic and experimental studies suggest potential effects such as disruptions in calcium signaling, oxidative stress and potential effects of cognition, neurodevelopment and reproductive health (such as impacts of fetal development, fertility and endocrine function). A major limitation across the literature is lack of standardized and objective exposure metrics by which epidemiological findings remain inconsistent. Many studies depend on self reported usage rather than objective measurements such as dosimetry or environmental monitoring. Furthermore, the distinction between near field and far field exposures complicate risk evaluation, as metrics like SAR does not adequately reflect cumulative and real life exposure scenarios. Although guidelines from ICNIRP effectively address established thermal effects of RF-EMF exposure, uncertainties remain regarding potential long term, low intensity and non thermal biological effects, particularly during sensitive developmental stages. The rapid development of wireless technologies, with 5G networks expanding across the globe and increasing more dependency on device at younger ages, means that there is urgent need to continuously re-evaluate current safety standards. Future research should focus on well designed studies, standardized exposure assessment especially those started during pregnancy and early life stages and adopt interdisciplinary strategies integrating experimental, epidemiological and computational modelling to better understand mechanisms and dose–response relationships. Further in future epidemiologic studies, more emphasis should be placed on the use of objective exposure assessment, standardised dosimetry, repeated exposure measurements, and rigorous control of potential confounding factors to improve causal inference. A prudent approach may therefore be considered in the form of precautionary measures such as minimizing unnecessary exposure, public awareness and promoting safe device practices. Ultimately, only rigorous, advanced research and reassessment will address existing knowledge gaps. This will inform evidence based regulatory policies for protecting the vulnerable populations in an increasingly wireless world.

## Glossary

RF-EMF: Radiofrequency Electromagnetic Fields
RF-EMR: Radiofrequency Electromagnetic Radiation
RFR: Radiofrequency Radiation
SAR: Specific Absorption Rate
ICNIRP: International Commission on Non-Ionizing Radiation Protection
IARC: International Agency for Research on Cancer
WHO: World Health Organization
ROS: Reactive Oxygen Species
DNA: Deoxyribonucleic Acid
MDA: Malondialdehyde
TOS: Total Oxidant Status
T-AOC: Total Antioxidant Capacity
SOD: Superoxide Dismutase
CAT: Catalase
GPx: Glutathione Peroxidase
GSH: Glutathione
NF-κB: Nuclear Factor Kappa B
MAPK: Mitogen-Activated Protein Kinase
ERK: Extracellular Signal-Regulated Kinase
JNK c-Jun: N-terminal Kinase
COX-2: Cyclooxygenase-2
TNF-*α*: Tumor Necrosis Factor Alpha
IL-6: Interleukin-6
LDH: Lactate Dehydrogenase
AST: Aspartate Aminotransferase
ALT: Alanine Aminotransferase
ALP: Alkaline Phosphatase
EEG: Electroencephalography
CNS: Central Nervous System
VGCC: Voltage-Gated Calcium Channels
TRPV1: Transient Receptor Potential Vanilloid 1
NO: Nitric Oxide
iNOS: Inducible Nitric Oxide Synthase
ONOO−: Peroxynitrite
HPO: axis Hypothalamic–Pituitary–Ovarian axis
GnRH: Gonadotropin-Releasing Hormone
LH: Luteinizing Hormone
FSH: Follicle-Stimulating Hormone
StAR: Steroidogenic Acute Regulatory Protein
3β-HSD: 3-beta Hydroxysteroid Dehydrogenase
17β-HSD: 17-beta Hydroxysteroid Dehydrogenase
E2: Estradiol
P4: Progesterone
AChE: Acetylcholinesterase
ChAT: Choline Acetyltransferase
DOPAC: 3,4-Dihydroxyphenylacetic Acid
5-HT: Serotonin
5-HIAA: 5-Hydroxyindoleacetic Acid
NMDA: N-Methyl-D-Aspartate
APAF1: Apoptotic Protease Activating Factor-1
PARP: Poly (ADP-ribose) Polymerase
OR: Odds Ratio
RR: Relative Risk
CI: Confidence Interval
SDQ: Strengths and Difficulties Questionnaire
CAKUT: Congenital Anomalies of Kidney and Urinary Tract
GSM: Global System for Mobile Communications
